# Molecular regulation of T-ALL cell infiltration into the CNS

**DOI:** 10.18632/oncotarget.21394

**Published:** 2017-09-30

**Authors:** Judy L. Cannon, Sreenivasa Rao Oruganti, Devan W. Vidrine

**Affiliations:** Judy L. Cannon: Department of Molecular Genetics and Microbiology & Department of Pathology University of New Mexico Health Sciences Center, Albuquerque, New Mexico, USA

**Keywords:** T-ALL, chemokine, carma1, CNS

Acute lymphoblastic leukemia (ALL) is a blood disorder arising from cells derived from either the T- or B-cell lineage. ALL is the most commonly seen cancer in pediatric patients, with T-cell acute lymphoblastic leukemia (T-ALL) accounting for nearly 20% of newly diagnosed ALL cases. Advances in therapy are improving event-free survival rates of T-ALL which exceed 80% in some clinical trials. However, central nervous system (CNS) infiltration by T-ALL cells and CNS relapse in pediatric T-ALL patients drive down survival rates, in some cases to as low as 40%. Current clinical practice addresses T-ALL CNS involvement with high-dose cranial radiation and intrathecal chemotherapy, resulting in adverse effects including neurotoxicity and secondary neoplasms. Successful management of CNS-implicated T-ALL disease remains problematic, largely because homing and survival mechanisms of leukemic cells into the CNS are unclear. Further, exactly which biomarkers drive CNS T-ALL disease remain unidentified. Current research efforts, including our own, seek potential molecular targets for blocking T-ALL disease in the CNS [1].

T-ALL arises from aberrant activation of a variety of signaling pathways that mediate cell growth, survival, and differentiation of thymocytes [2]. Notch1 is a major transcription factor driving T-cell development, and accumulated mutations leading to constitutive activation of the Notch1 pathway are linked to more than half of T-ALL cases. Animal models using oncogenic Notch1 expression to investigate mechanisms of T-ALL disease have been useful to understand the molecular basis of T-ALL, providing therapeutic targets [1, 3]. Recent findings suggest molecules crucial for T-cell receptor (TCR) signaling, like ZAP70 and PKCθ, can play a role in T-ALL pathogenesis [4]. Downstream of ZAP70 and PKCθ, TCR signaling activates Carma1, Bcl10, and MALT1, collectively known as the CBM complex, resulting in NF-κB activation [5]. Constitutive activation of CBM signaling is now recognized as a common feature of an increasing number of B- and T-cell malignancies [5].

We tested the role of Carma1 in T-ALL leukemogenesis and found that Carma1 regulates T-ALL CNS disease [1]. Absence of Carma1 leads to increased survival in T-ALL using multiple animal models of T-ALL. Strikingly, Carma1 expression controls migration of T-ALL cells into the CNS. Histological examination of CNS tissue in animals with Carma1-deficient T-ALL cells shows little to no signs of leukemic cell infiltration into the CNS. This is in contrast to WT T-ALL cells which show significant accumulation in the CNS. Further, in agreement with existing data that correlates increased expression of Carma1 with T-ALL disease, we find that CNS-positive T-ALL patients express significantly higher Carma1 in comparison with T-ALL patients that are CNS-negative at the time of diagnosis [1]. Our study highlights the essential role of Carma1 in T-ALL disease, particularly in driving CNS infiltration of T-ALL cells. These results suggest Carma1 inhibition is a potential therapeutic strategy to control T-ALL CNS disease.

In addition to Carma1, other groups have demonstrated a role for the chemokine receptor CCR7 in trafficking of leukemic T-cells into the CNS [3]. CCR7 typically mediates naïve T-cell migration to lymph nodes, yet its presence on T-ALL cells decreases survival of animals with T-ALL. Interestingly, our research finds that CCR7 and Carma1 co-localize in T-ALL cells, and Carma1 expression is important in migration of leukemic T-cells in response to the CCR7 ligand CCL21 [1]. While our data suggests that CCR7 may be upstream of Carma1 in driving T-ALL cell migration to the CNS, it is also possible that CCR7 and Carma1 regulate T-ALL cell migration independently of one another. Besides CCR7, other chemokine signaling pathways including CXCR3 and CXCR4 have been shown to play a role in initiation and progression of T-ALL disease [3, 6, 7]. How CCR7 and other chemokine receptors interact with signaling pathways such as Carma1 in regulating CNS disease in T-ALL remains to be clarified.

Movement of immune cells through the blood-brain barrier into the CNS is a decades-old topic of interest, yet much is left to discover. Identifying molecules that drive leukemic T-cells to the CNS has implications not only for T-ALL, but diseases like multiple sclerosis, in which lymphocyte trafficking to the CNS is a central issue. Several targetable pathways including Notch1, IL-7/IL-7R, PI3K/Akt, and MAPK are associated with T-ALL progression [8], but the role for these pathways in T-ALL CNS migration thus far remains unexplored. Future investigations into molecular determinants of T-ALL CNS migration hold promise for developing novel therapies to specifically target T-ALL CNS disease.
